# Tools, methods, and applications for optophysiology in neuroscience

**DOI:** 10.3389/fnmol.2013.00018

**Published:** 2013-07-17

**Authors:** Niklas Smedemark-Margulies, Josef G. Trapani

**Affiliations:** Department of Biology and Neuroscience Program, Amherst CollegeAmherst, MA, USA

**Keywords:** ChR2, optical reporter, genetically encoded sensors, optogenetics, halorhodopsins, channelrhodopsin, photosensors, photoactuators

## Abstract

The advent of optogenetics and genetically encoded photosensors has provided neuroscience researchers with a wealth of new tools and methods for examining and manipulating neuronal function *in vivo*. There exists now a wide range of experimentally validated protein tools capable of modifying cellular function, including light-gated ion channels, recombinant light-gated G protein-coupled receptors, and even neurotransmitter receptors modified with tethered photo-switchable ligands. A large number of genetically encoded protein sensors have also been developed to optically track cellular activity in real time, including membrane-voltage-sensitive fluorophores and fluorescent calcium and pH indicators. The development of techniques for controlled expression of these proteins has also increased their utility by allowing the study of specific populations of cells. Additionally, recent advances in optics technology have enabled both activation and observation of target proteins with high spatiotemporal fidelity. In combination, these methods have great potential in the study of neural circuits and networks, behavior, animal models of disease, as well as in high-throughput *ex vivo* studies. This review collects some of these new tools and methods and surveys several current and future applications of the evolving field of optophysiology.

## BACKGROUND

### INTRODUCTION

Experiments using voltage-sensitive chemicals to optically monitor activity in neurons have been evolving for several decades (Woolum and Strumwasser, 1978). In recent years, there has been a dramatic increase in the use of genetically encoded proteins to visualize neuronal activity *in vivo* (Akerboom et al., 2012; Jin et al., 2012). In addition, the field of optogenetics, which uses genetically encoded proteins for optical control of cellular functions, is growing rapidly (Fenno et al., 2011). While there are also many photo-responsive chemicals used in optophysiology, here we will primarily review the use of genetically encoded proteins. Discussion will encompass proteins with activity-sensitive fluorescence or luminescence (photosensors), optogenetic proteins (photoactuators), methods for both optical manipulation and measurement of these photo-sensitive proteins, and the application of these proteins in physiologically relevant research. Thus, this review will discuss optophysiology as a field that includes engineered proteins, the molecular biology components for their expression, and the equipment, and software necessary for their use in neuroscience. Finally, we will review several successful applications of optophysiology for clinically relevant research on animal models of disease and high-throughput *ex vivo* drug screens.

### OPTOPHYSIOLOGY MOVING FORWARD

Traditionally, examining the spatiotemporal characteristics of neuronal activity has been accomplished using electrophysiological techniques. Electrophysiology is the prevalent approach to measuring and manipulating neuronal activity in experiments such as cellular stimulation combined with field recordings for functional analysis of specific brain regions (Salah and Perkins, 2011), inducing long-term potentiation and depression of neuronal activity (Pavlowsky and Alarcon, 2012), and observing sensory encoding of physiologically relevant stimuli (Trapani et al., 2009). Electrophysiological experiments can combine data from multiple electrodes (Xu et al., 2012), can integrate electrodes into a neuronal culture to facilitate prolonged measurements (Regehr et al., 1989), and can even combine simultaneous electrophysiology with optophysiology (Bender and Trussell, 2009). These creative techniques highlight the power of modern electrophysiology.

There are some limitations inherent in electrical stimulation and recording with standard electrophysiological techniques. For instance, it is difficult to control an entire neuron’s membrane potential from one point of injected current (Spruston et al., 1993). Furthermore, the placement of the electrode determines the neuron to be examined, often making it technically challenging to control a population or subset of specific neurons. This limitation often precludes the number and type of experiments that can be performed *in vivo*. In contrast, by exploiting genetics, optophysiology can target specific cells or tissues to achieve control of an individual cell or a population of cells *in vivo*. In addition, the proteins used for optophysiology are frequently tagged with a marker to track the location of expression within a cell or brain region. Thus, the nature of genetically encoded optical probes and actuators allows for spatiotemporal control of expression across cellular populations and can be further managed through optically focused activation. Furthermore, optophysiology has the ability to survey large brain regions in real time with minimal disruption to the organism (Ackman et al., 2012).

There are also important points for consideration of optophysiology. First, optimizing the action spectra and kinetics of proteins for specific applications may require screening for useful mutations or creating recombinant isoforms (Gunaydin et al., 2010; Akerboom et al., 2012, 2013). Similarly, achieving appropriate expression patterns and subcellular location can be labor intensive and expensive. Also, high levels of transgenic protein may impair cellular function over time even with appropriately controlled protein localization (Miyashita et al., 2013). Next, the reliance on light necessitates the optical accessibility of the tissue and cells being studies. Finally, optophysiology requires specialized optics and imaging technology for collection and analysis of small, fast signals – ideally with low background noise.

## OPTOPHYSIOLOGY TOOLS

### PHOTOSENSORS

The key principal of an optical sensor is that it exhibits a change in fluorescence or luminescence following a conformational response to changes in its chemical, mechanical, or electrical environment, thus providing an optical report of this change (for a list of popular genetically encoded photosensors see **Table 1**). For example, detection parameters can include the concentration of a ligand of interest, the pH of the intracellular or extracellular environment, or neurotransmitter vesicle secretion (Miesenböck et al., 1998). As is true for photoactuators (discussed below), an ideal photosensor should be highly sensitive to the cellular function of interest and specific enough to minimize background, non-specific fluorescence. The sensor should also be minimally invasive and not disrupt normal cellular function. In most cases, it is desirable to maximize the speed of a sensor’s response cycle so that fast cellular activity may be visualized. Fluorescent photosensors ought to be specifically sensitive at their peak excitation wavelength, which may be optimized for spectral separation from other photosensitive proteins that could be concomitantly expressed (Akerboom et al., 2013).

**Table 1:** Selected examples of genetically encoded photosensors.

Sensor	Protein components	Peak excitation wavelength (nm)	Parameter measured	Function reported	Reference
GCaMP, GCaMP5, GCaMP7	M13, cpEGFP, CaM	489	Calcium	Ca^2^^+^ concentration (e.g., intracellular stores or VGCCs)	Nakai et al. (2001); Pologruto et al. (2004), Akerboom et al. (2012); Muto et al. (2013)
Cameleon	M13, ECFP, EYFP, CaM	440 ECFP	Calcium	Ca^2^^+^ concentration (e.g., intracellular stores or VGCCs)	Truong et al. (2001, 2007)
Clomeleon, Cl^-^ Sensor	CFP,TFP	440 CFP	Chloride	Cl^-^ concentration (e.g., via GABA receptors)	Kuner and Augustine (2000) Markova et al. (2008)
ArcLight	ciVSP, super ecliptic pHluorin	490	Voltage	Action potentials, neurotransmission-mediated ion channel activity	Jin et al. (2012)
ElectricPk	ciVSP, cpEGFP	488	Voltage	Action potentials, neurotransmission-mediated ion channel activity	Barnett et al. (2012)
Arch	Archaerhodopsin, EGFP	640 (arch), 488 (GFP)	Voltage	Action potentials, neurotransmission-mediated ion channel activity	Kralj et al. (2012)
pHlourin	GFP	470	pH	Vesicle fusion using synapto-pHlourin	Miesenböck et al. (1998) Bowser and Khakh (2007)
SuperGluSnFr	GltI, ECFP, Citrine	476 CFP	Glutamate	Glutamate concentration (e.g., neurotransmitter release)	Hires et al. (2008)
Aequorin	Apoaequorin, coelenterate luciferin	n/a; luminescent	Calcium	Ca^2^^+^ concentration (e.g., intracellular stores or VGCCs)	Prasher et al. (1985)

*
A sample of popular genetically encoded protein photosensors. M13, M13 domain from myosin light chain kinase; cpEGFP, circularly permuted enhanced GFP; CaM, Calmodulin; ciVSP, *C. intestinalis* voltage-sensitive phosphatase; VAMP2, vesicle-associated membrane protein 2; pHlourin, mutant, pH-sensitive GFP; GltI, glutamate periplasmic binding protein; ECFP, enhanced cyan fluorescent protein; FRET, Förster resonance energy transfer.*

Many fluorescent photosensors rely on a single fluorophore, while others have a pair of fluorophores that exhibit Förster resonance energy transfer (FRET) upon activation of one of the molecules by a photon of a particular wavelength (or two of double the wavelength in the case of two-photon excitation). For single fluorescent molecules, the efficiency of fluorescence provides a readout of changes in the fluorophore’s local environment. For FRET pairs, changes in the chemical environment affect the efficiency of energy transfer between the two fluorophores, which is then measured as a change in the ratio of their individual fluorescences. This ratiometric imaging is beneficial because it relies on changes in relative fluorescence and not absolute fluorescence levels. Since background noise such as non-specific autofluorescence may not affect FRET, ratiometric imaging may be less problematic and data can often be more easily compared across different experiments (Tadross et al., 2009; Kindt et al., 2012). In addition to genetically encoded sensors, there are also photoresponsive chemicals (e.g., Fura-2) that are used in optophysiology due to their favorable response characteristics (Dawitz et al., 2011).

Many cellular properties may serve as functional indicators of activity, two of which are membrane potential and intracellular calcium concentration. In the past, the limited availability of voltage-sensitive proteins that exhibit fast, large-magnitude changes in photochemical properties (within the narrow ~100 mV range of an active neuron’s membrane potential) made calcium-sensitive probes a more frequent choice for monitoring cellular activity. Changes in intracellular calcium concentration have a clear relevance to synaptic function and serve as an indicator for pre- and post-synaptic neuronal activity. As a result of the functional link between changes in calcium concentration and neuronal activation, genetically encoded calcium indicators (GECIs) are a popular choice for optically monitoring neuronal activity. Such measurements can be performed at both the single cell and whole-brain levels (Ackman et al., 2012; Akerboom et al., 2012).

Researchers seeking calcium-responsive proteins for optical probe design have exploited the key role of calcium in signaling cascades. For example, the calcium-binding messenger protein, calmodulin (CaM) has been used to create a chimeric GECI called GCaMP. The GCaMP molecule comprises a circularly permuted green fluorescent protein (GFP) bound to both the calcium binding domain of calmodulin and the calmodulin-binding domain of a myosin light chain kinase (M13; Nakai et al., 2001). The fusion protein exhibits efficient fluorescence when calcium is bound, whereas its fluorescence decreases sharply when calcium is not bound. GCaMP has been successfully used to visualize increases in intracellular calcium concentration that result from influx through voltage-gated calcium channels and/or calcium permeable receptors, or release from intracellular stores. Efforts to improve the GCaMP probe are ongoing. Mutant forms have been developed with increased signal to noise ratios such as GCaMP5 (Akerboom et al., 2012), and one of the latest iterations, GCaMP7, is both fast and sensitive enough to monitor neuronal activity of the zebrafish optic tectum in real time (Muto et al., 2013).

Ratiometric indicators may be favorable when noisy data is expected, since background activity may have a similar impact on the detection of each fluorophore in the pair. For example, Cameleon is a chimeric, ratiometric calcium indicator, with the same calmodulin and M13 domains as GCaMP and a pair of fluorophores that exhibit FRET. Upon calcium binding, there is an increase in FRET efficiency, which provides a ratiometric measure of calcium changes (Truong et al., 2001, 2007). Aequorin is an example of a calcium-sensitive luminescent protein; it emits a blue photon upon binding of calcium (Dugué et al., 2012). This molecule avoids the potentially deleterious effects of photo-excitation. However, since this molecule is luminescent rather than fluorescent, emission intensity cannot be directly increased through greater intensity of photo-stimulation.

Similary, a ratiometric indicator of chloride concentration also exists (Clomeleon; Kuner and Augustine, 2000) and was recently improved to increase its chloride sensitivity into the physiological range (Cl-Sensor; Markova et al., 2008). A very recent study by Batti et al. used this ratiometric probe to generate Cl-Sensor transgenic mice (Batti et al., 2013). In this study, the authors use electrophysiology to calibrate chloride-dependent fluorescence changes to the intracellular chloride concentration in both hippocampal and cortical neurons in brain slices. They further show that non-invasive monitoring of Cl-Sensor fluorescence can report the changes in intracellular chloride that occur during epileptiform activity (Dzhala and Staley, 2003; Ben-Ari et al., 2012). This study provides a fine example of how electrophysiology and optophysiology can be used concurrently to complement and validate each other.

Voltage probes were originally more difficult to use in part because early examples tended to show a small fluorescence response across the physiological range of membrane potential (Barnett et al., 2012; Mutoh et al., 2011). Voltage sensors couple the movement of a dipole in the membrane to the conformation of a fluorophore or FRET pair to produce a detectable change in fluorescence. Ideally, the sensitivity and speed of fluorescence change would allow the researcher to track the time course of action potentials and other transient ionic currents that change the membrane potential. Voltage sensitive proteins (VSPs) have several potential advantages over calcium photosensors. Since they do not sequester calcium, these sensors may be less likely to disrupt *in vivo* activity compared to a calcium-binding probe (Ouanounou et al., 1999). In theory, voltage sensors can also detect non-calcium-dependent sub-threshold activity and activity at gap junctions where neuronal activity may not involve calcium (Perron et al., 2012). This feature allows researchers to better examine integration of neuronal synaptic inputs. As voltage probes also show activity throughout the cell rather than at regions of localized calcium influx or release from intracellular stores, they may produce a signal that more faithfully follows the activity of the neuron. An example of this is seen with voltage-sensitive chemicals, which were used to track the initiation and movement of an action potential along an axon (Foust et al., 2010). A current genetically encoded voltage sensor with great potential is Archaerhodopsin-3 (Arch). This prominent voltage indicator protein is derived originally from a microbial proton pump. Modifications made to the protein by the Cohen lab have inactivated the proton pump, but maintained its favorable voltage response characteristics and kinetics (Kralj et al., 2012). Another fluorescent voltage sensor called ArcLight was recently developed, and since its fluorescence arises from a very different fluorophore, it has a distinct set of properties as compared to Arch (Jin et al., 2012).

### PHOTOACTUATORS

In early experiments on photo-activation of neurons, a genetic component allowed for cell-type specificity, but exogenously applied photoactuator chemicals were required to elicit responses (Zemelman et al., 2003). This external chemical modification limited early approaches to *in vitro* experimentation and often constrained experiments due to the toxicity or functional lifespan of the organic chemical. Ideally, a genetically encoded protein could serve many of the same functions as systems requiring exogenous actuators while avoiding many of their limitations. The first “optogenetic” photoactuator to satisfy these criteria came in the form of a light-gated proton channel, channelrhodopsin (ChR1), isolated from the photosynthetic algae *C. reinhardtii* (Nagel et al., 2002). Subsequent modification of channelrhodopsin created channelrhodopsin-2 (ChR2) with a peak excitation wavelength of 470 nm, and increased conductance to cations that allows for the depolarization of cells upon photo-activation (Nagel et al., 2003). Initial experiments revealed that physiologically relevant non-invasive activation of hippocampal neurons could be achieved through ChR2 expression (Boyden et al., 2005). More recently, mutagenesis screens and knowledge-guided structural mutations have led to many variants of the channel that differ in ion conductance, response spectrum and response kinetics (Akerboom et al., 2012; Rein and Deussing, 2012; Muto et al., 2013).

To make more use of the specificity of gene targeting, one of the common goals of developing new optogenetic photo-actuator proteins is to modify the function of an existing photoactuator to perform a very specialized function. For most applications, the ideal photoactuator is highly sensitive so that stimulation intensity may be minimized and a controlled response will be observed with minimal disruption to cellular function. Additionally, photoactuators are often optimized in their kinetics or activation spectrum for a given experiment. For instance, available photoactuators include those capable of high-frequency signal transduction (Gunaydin et al., 2010) or toggle-switch activity where the actuator can be turned on *and* off (Berndt et al., 2009), and current efforts at spectral modification are focusing on proteins that are spectrally divergent from other optogenetic tools to allow for combined expression with minimal overlap of activation wavelength (Zhang et al., 2008). In the next few paragraphs, we will discuss some of these newly developed or modified optogenetic actuators.

Several groups have developed channelrhodopsin variants that circumvent specific limiting characteristics of ChR2. Gunaydin et al. (2010) introduced two point mutations in the channel that allow for photostimulation up to a frequency of 200 Hz while also increasing the response fidelity of the protein. These modifications make for a protein with much better temporal precision, permitting experiments probing the importance of high-frequency bands such as gamma activity (Gunaydin et al., 2010). Berndt et al. (2009) also found two point mutations in the original ChR2 sequence that dramatically increase the time constant for channel closing, leading to a more stable open-state after photo-stimulation. Importantly, the researchers were able to achieve de-activation through green light stimulation, resulting in a channel that can be photo-toggled on and off (Berndt et al., 2009). Since multi-component systems tend to exhibit spectral overlap between proteins, the stable “on” state of this actuator provides an appealing way to minimize noise from non-specific inputs. Zhang et al. (2008) describe a channelrhodopsin protein derived from *V. carteri*, VChR1, with an activation spectrum peak that is red-shifted by about 70 nm as compared to that of ChR2. This spectral separation provides the possibility for simultaneous, independent control of multiple cell types by photostimulation at different wavelengths (Zhang et al., 2008). A wide range of additional combinations between various ChR2 mutants have been explored to optimize a photoactuator for a given experimental setup (Yizhar et al., 2011). The success of these efforts to modify the properties of channelrhodopsin demonstrates the potential for protein engineering to produce photoactuators precisely suited for an application of interest.

A wide variety of other light-gated ion channels and pumps have also been developed or isolated (see **Table 2**). *N. pharaonis* halorhodopsin (NpHR), a chloride ion pump activated by 570 nm light, provides the crucial inhibitory counterpart to the stimulatory channelrhodopsin (Zhang et al., 2007). Similar to channelrhodopsin, NpHR has been improved by molecular engineering, especially to improve its subcellular localization (eNpHR3.0; Gradinaru et al., 2008). Proton pumps like bacteriorhodopsin can achieve a similar inhibitory effect (Dugué et al., 2012). These recombinant photoactuators provide control of neuronal membrane potential, but there are also many experimentally interesting optogenetic targets in cellular metabolism and metabotropic activity.

**Table 2:** Selected examples of optogenetic photoactuators.

Photoactuator	Source organism	Peak excitation wavelength (nm)	Parameter affected	Functional purpose in neurons	Reference
Step function opsins	*C. reinhardtii*	470 on, 530 off	Cation conductance	Toggle of Vm	Berndt et al. (2009)
VChR1	*V. carteri*	589	Cation conductance	Depolarization of Vm	Zhang et al. (2008)
Bacteriorhodopsin	*Halobacteria*	568	Proton pumping	Hyperpolarization of Vm	Nishikawa et al. (2005)
ChR2 (channelrhodopsin)	*C. reinhardtii*	470	Cation conductance	Depolarization of Vm	Nagel et al. (2003)
ChETA	*C. reinhardtii*	470	Cation conductance	High frequency depolarization	Gunaydin et al. (2010)
LiGluR	Vertebrates	380	Cation conductance	Depolarization of Vm	Volgraf et al. (2006); Li et al. (2012)
chARGe	*D. melanogaster*	430-550	PLC-mediated cation conductance	Depolarization of Vm	Zemelman et al. (2002)
NpHR (halorhodopsin)	*N. pharaonis*	570	Chloride conductance	Hyperpolarization of Vm	Zhang et al. (2007)
Opto-α_1_AR	Chimeric *B. primigenious* and *H. sapiens*	504	IP_3_, DAG generation	Gq/PLC signaling pathway	Airan et al. (2009)
Opto-β_2_AR	Chimeric *B. primigenious* and *M. auratus*	504	cAMP generation	Gs/AC signaling pathway	Airan et al. (2009)

*Here we list optogenetic actuators that have been developed from different source organisms, with efforts made to vary the absorption spectra, emission spectra, and target function. Vm, membrane potential; NDBF, Nitrodibenzofuran; IP_3_, Inositol 1,4,5-triphosphate; DAG, diacylglycerol; cAMP, cyclic adenosine monophosphate; Gq/PLC, phospholipase-C; Gs/AC, adenylyl cyclase; ROS, reactive oxygen species; α_1_AR, alpha-1 adrenergic receptor; β_2_AR, beta-2 adrenergic receptor.*

So-called “optoXRs,” chimeric or recombinant photo-inducible G protein-coupled receptors, allow intervention in metabotropic signaling pathways. As in other areas of the optogenetic field, optoXR design has progressed rapidly. Miesenböck and colleagues formulated a system called “chARGe” in 2002 that requires transfection of *Drosophila* Arrestin-2, rhodopsin, and the appropriate G-protein (Zemelman et al., 2002). Subsequently, Deisseroth and colleagues designed a simpler optoXR composed of a single-component chimeric receptor from a bovine rhodopsin and the intracellular G-protein-interacting domains of an adrenergic receptor (see **Table 2**; Airan et al., 2009).

Another source of optogenetic actuators takes advantage of native, ligand-gated receptors. Clever engineering can allow a receptor to be photosensitive while also retaining its native ligand sensitivity. An example of this technique is seen in the ionotropic glutamate receptor, which was modified with a photo-switchable, tethered ligand allowing for light-activation of the resulting photoactuator (LiGluR; Volgraf et al., 2006). Many successful experiments with LiGluR have been done using zebrafish, where its transgenic expression has been used to study motor circuits and behavior (reviewed in Simmich et al., 2012).

### OVERVIEW OF TRANSGENIC STRATEGIES

The list of photosensors and photoactuators with new and interesting properties continues to grow, as do the techniques for effective transgene delivery. Due to their relative cost effectiveness and simplicity, the use of viral vectors, such as adeno-associated viral (AAV) vectors and lentiviral vectors for transgene delivery is quite common (Cooray et al., 2012). Expression can be controlled through copy number or the use of specific promoters and enhancers. Some cell specificity can be achieved by selecting a virus with appropriate tropic bias or by pseudotyping – repackaging the genetic material of one virus in the protein capsid of another to produce a combination viral particle that will readily prefer to infect a researcher’s target cell type (Wang et al., 2011). Furthermore, spatial targeting of viral transfection may be achieved by stereotaxic injection and post-hoc confirmation. Importantly, the location of genomic integration of virally delivered genes may be random, and unwanted mutagenic effects or unexpected gene silencing can hinder this approach. In zebrafish, this non-specific effect can be avoided with transgene expression using injection of plasmid vectors (Faucherre et al., 2009), and can include genomic integration using meganuclease-mediated gene insertion (Grabher et al., 2004) or tol2 recombination (Suster et al., 2009).

Stable transgenics, where the transgene is integrated into the host’s germline, offers several advantages including the ability to repeat experiments across animals or ensure comparability between experiments on different organ systems and brain regions. In addition, if non-targeted gene insertion will cause unwanted effects, researchers can target transgene insertion through techniques including homologous recombination (von Koch et al., 1997). This may help to minimize transgene silencing or mutagenic effects due to insertion locus. Also, stable integrated transgenic organisms may show greater homogeneity of expression levels across tissues and animals, and comparisons can be made between different developmental time points. In order to exploit the expression pattern of a well-characterized promoter, homologous recombination can be used to insert a gene downstream of an endogenous promoter, or the inserted sequence may itself contain a promoter region.

### MODULAR TRANSGENICS FOR OPTOPHYSIOLOGY

**FIGURE 1:**
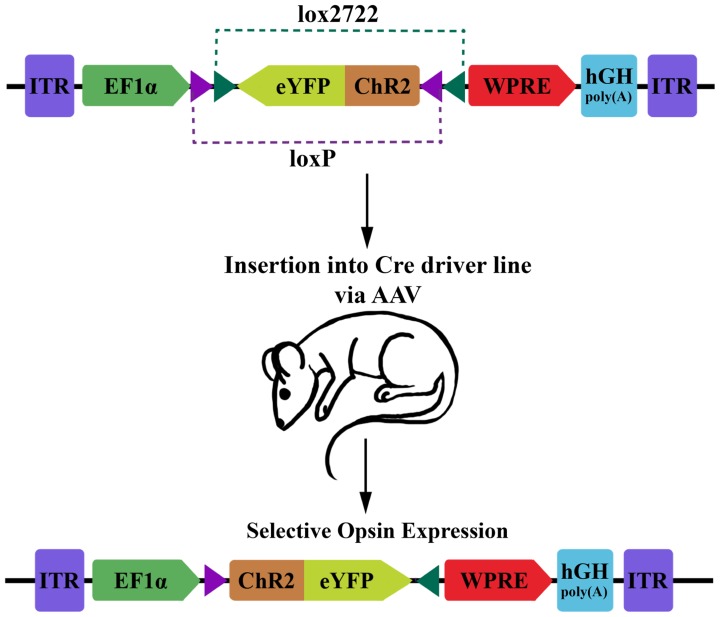
**Diagram of DIO transgenic system in mice.** The double-floxed inverse open reading frame (DIO) construct comprises inverted terminal repeats (ITR), the EF1α promoter, an eYFP-ChR2 fusion gene surrounded by a pair of LoxP sites and a pair of Lox2722 sites oriented inward, a woodchuck hepatitis virus post-transcriptional regulatory element (WPRE) and a human growth hormone polyadenylation signal (hGH polyA). The eYFP-ChR2 gene starts in an inverted, inactive orientation. Expression of Cre recombinase will cause serial recombination resulting in the active, fixed orientation of the transgene (bottom; figure adapted from Sohal et al., 2009).

For optophysiology with genetically encoded proteins, specific over-expression is generally very important. This is both for ensuring adequate activation when using photoactuators, and for increasing the signal-to-noise ratio of experiments with photosensors. Several modular transgene systems have been developed to facilitate cell-type specificity and strong expression. The double-floxed inverse open reading frame (DIO) strategy devised by Karl Deisseroth’s group provides one such method for minimizing so called “transcriptional leakage” while simultaneously achieving high expression levels (**Figure 1**; Sohal et al., 2009). This strategy improves on a common two-part scheme for transgene expression, wherein a promoter with high cell-specificity drives the expression of Cre recombinase, while a promoter with stronger expression drives a desired transgene with a floxed stop codon. In this traditional formulation of the floxed-stop scheme, the researcher can combine the spatiotemporal specificity of the first promoter with the transcriptional strength of the second. Excision of the stop codon and subsequent expression of the transgene will ideally occur only following Cre expression at the appropriate developmental time point or within cells of interest. Practically speaking, though, there can be transcriptional leakage when the stop codon fails to completely prevent transcription of the transgene. This can be particularly detrimental in the case of a high-expression promoter that is generally ubiquitously expressed and constitutively active. The innovation of Sohal et al.’s (2009) modification is that the transgene is initially inverted and truly inactive in its “off” state. Expression is accomplished using two pairs of incompatible lox sites that flank the inverted transgene. Serial recombination will first reorient the transgene and then excise one of the two lox sites from each pair, and the final state of the gene contains only an incompatible pair of lox sites that are no longer responsive to Cre. This reorientation mechanism can be used as a Cre-on system where the initial orientation of the gene is inactive, or Cre-off where the initial orientation of the gene is active. To add even more degrees of control, this mechanism can be used as a Cre-switch system if the double-floxed region contains two genes in opposite orientations with a stop codon between, though transcriptional leakage of the stop codon again becomes a factor in this setup (Saunders et al., 2012).

Another method for creating modular transgenics was developed by Tanaka et al. (2012). Called the KENGE-tet system (short for knock-in mediated enhanced gene expression) this system is based on the tetracycline transactivator-responsive TetO promoter and is comprised of two parts, the transgenic protein of interest (e.g., ChR2) and a tetracycline transactivator gene (Tanaka et al., 2012). Like the DIO strategy, this approach allows the researcher to separately control several aspects of expression pattern. The promoter driving the gene coding for the tetracycline transactivator (tTA) determines the tissue specificity of gene expression, while the expression intensity and timing are controlled by the exogenous administration of tetracycline to either trigger (Tet-on) or silence (Tet-off) expression of the transactivator. To prevent unwanted chromosomal silencing or suppression effects, Tanaka et al. (2012) chose to insert the transgene driven by the TetO promoter adjacent to the beta-actin gene, known for its ubiquitous high-level expression.

These two modular expression systems offer a variety of scalable expression patterns in transgenic animals. New combinatorial possibilities can be created by crossing existing driver lines of tTA or Cre animals with new transgenic protein lines, or vice versa. This also enables one to exploit the many databases of driver lines such as the GENSAT project, which pre-compile driver lines with physiologically interesting expression patterns (e.g., Geffers et al., 2012). Often such databases of driver lines are constructed using an enhancer trap screen. In this approach, a large number of transgenic animals are generated through random insertion of a marker gene. The resulting expression patterns of the marker gene are screened, allowing the researcher to select a pattern appropriate for the cell population or for the developmental time course they aim to modify (Asakawa and Kawakami, 2009). By sequencing the genomic DNA upstream or downstream from the integrated marker, the researcher can identify the genetic region that is likely to be driving a particular expression pattern. Thus, an organism with an interesting expression pattern can be subsequently used to obtain a desired promoter or enhancer for driving a transgene of interest (see, for example, Pujol-Martï et al., 2012). This technique lends itself well to use with the Gal4/UAS transcriptional system (Scott et al., 2007; Asakawa and Kawakami, 2009). Here, the Gal4 gene product activates transcription of a transgene located after the cognate upstream activator sequence (UAS), which confines expression to the area of tissue-specific GAL4 expression. Thus, by generating many GAL4 driver lines and a library of UAS-transgene lines, offspring with a desired transgenic expression pattern can be produced by the proper mating combination.

Another approach to improving population-selective expression of transgenes is repression of transcription in undesired tissues. The transcription factor REST is expressed in non-neuronal tissues and has been shown to repress inappropriate transcription of neuronal genes by binding to a DNA motif called the neuron-restrictive silencing element (NRSE; Mandel et al., 2011). In order to probe the circuit function of neurons by ablation, Bergeron et al. (2012) developed a derivative silencing element called REx2. They validated and quantified the effect of this silencing element by showing that restriction is relaxed by the introduction of a morpholino (a small oligonucleotide that blocks translation of an mRNA target) against the REST gene. Importantly, this silencing motif can be used in conjunction with existing promoter regions derived from enhancer trap screens to further improve specificity. The authors show the impact of their silencing element particularly in the context of ablation experiments, though it also has broad applicability to marker genes or other functionally diverse transgenes (Bergeron et al., 2012). In summary, there are many approaches available for expression of genetically encoded photosensors and photoactuators. It is now up to the optophysiologist to determine the most appropriate method for their desired experiment and animal model system.

### OPTICS HARDWARE AND SOFTWARE

Development and adaptation of optics technology is a key component for progress in optophysiology. Moving from excitation of fluorophores using wide-field illumination to true cell-specific excitation has required not only advances in transgene expression targeting, but also strategic application of knowledge from fields including optics and multi-photon laser microscopy.

For spatial targeting the activation of photoactuators, one approach is the use of digital micro-mirror devices (DMDs). These devices produce a variable spatial light pattern with a resolution limit set by the surface pixilation of a given DMD. The DMD operates by changing the angle of reflection in areas corresponding to the desired “dark” regions on a sample such that the beam path from these points will not reach the target. In this way, they are subtractive, meaning that there will necessarily be a loss of stimulation power when the DMD is in use, which may require a compensatory increase in the initial light power (Oron et al., 2012).

Another approach for targeting excitation of a photoactuator involves the use of spatial light modulators (SLMs; Nikolenko et al., 2008; Oron et al., 2012; Prakash et al., 2012). SLMs are an instance of digital holography, a technique that uses phase-modifying filters to produce a cross-sectional pattern of phase relationships. This pattern of transmitted light, when focused onto the plane of interest, will undergo constructive interference where activation is desired and destructive interference where activation is not desired. The technique allows a researcher to avoid regions of overlap with non-target cells and thereby maintain cell-specific activation. Packer et al. demonstrated the possibility of using this technique to achieve a three-dimensional pattern of activation by simultaneously but independently stimulating cells in different focal planes (Packer et al., 2012). This technique is still under development, and there currently remain limits on the degree of spatial flexibility afforded by SLMs.

Recently, Kim et al. (2013) described a new cellular-scale optoelectronic implant that allows remote control of cellular activity in freely moving animals. Such advancements in optics technology open up the potential to explore the role of circuit elements in an awake behaving animal.

The technology necessary for optical recording differs from the technology necessary for optical activation. For some applications, wide-field light may be sufficient for imaging photosensor responses. Here, the emphasis would shift to designing cameras and software of sufficient speed and resolution to detect events that may occur up to the kilohertz range and also to detect faint signals in an optically noisy environment (Rózsa et al., 2007). For *in vivo* systems or for achieving greater light penetration, stimulation through fiber optic cables may be preferable (Ung and Arenkiel, 2012). Some research groups have begun using even more sophisticated approaches. Ahrens et al. (2013) recently made dramatic progress in scaling up optics technology for use with physiological systems, designing a system capable of imaging 80% of neurons in a larval zebrafish brain *in vivo* at 0.8 Hz. Their technique used scanning light-sheet fluorescence microscopy with a GCaMP photosensor protein. In achieving such large-scale activity measurements, they have provided a crucial proof of concept for the future of whole-brain research, and demonstrated the potential of currently existing protein photosensors and photoactuators (Ahrens et al., 2013).

Inevitably, the limits of currently available optics equipment will have to be addressed by the use of software for tasks like interpolation between time-series measurements when image acquisition speed is insufficient, or screening out unrelated activity when background noise is high. Development of this software is as important for making meaningful conclusions as the technology necessary for data collection. Key issues to be addressed by any software system include noise filtration, image alignment (especially for ratiometric and time lapse imaging), compensation for movement artifacts, defining discrete signals and assigning those signals to a source cell, and statistical analysis of signals to draw meaningful conclusions. Cutting-edge video processing techniques may facilitate data acquisition. For instance, a group at MIT’s CSAIL recently published open-source code for Eulerian Video Magnification that greatly amplifies small visual signals for easier detection (Wu et al., 2012).

## PHYSIOLOGICAL APPLICATIONS

### BEHAVIOR

Optophysiology has been applied to a diverse range of animal models in order to elicit behavioral responses. The conclusions that have been drawn vary as much as the experimental model and question. In 2005, Lima and Miesenböck expressed ligand-gated ionotropic P2X purinoreceptor in giant fiber neurons implicated in escape reflex initiation in the fruit fly. This study showed that decapitated flies still had normal escape responses to giant fiber activation by photo-uncaging a modified version of ATP (Lima and Miesenböck, 2005). This early and dramatic demonstration of behavioral control through optical activation of neurons was an important proof of concept for the field. Leifer et al. provided sharp contrast for the pace of progress in 2011 with the development of a system called CoLBeRT capable of delivering stimulation pulses at 50 Hz with a spatial resolution of 30 μm. Using this technique, they interacted with muscle cells and touch receptor cells to manipulate the movements of a freely swimming worm (Leifer et al., 2011).

Witten et al. (2010) investigated the role of cholinergic interneurons in the nucleus accumbens (NAc) using AAV vector delivery of Cre recombinase-dependent eNpHR3.0. Despite the low proportion of these interneurons in the NAc, the researchers found that inhibition of their activity by photo-activation of eNpHR3.0 during a cocaine place-conditioning paradigm caused a significant decrease in acquired, conditioned place preference (Witten et al., 2010). In contrast to previous ideas about NAc circuit composition, these results indicated that the NAc cholinergic cells are important to reward based learning. Importantly, this study also showed the power of optophysiology for behavioral studies.

More recently, Liu et al. (2012) investigated the network correlate of a memory within the dentate gyrus circuitry of the hippocampus in mice. Using a novel technique for cell activity-dependent expression of ChR2, the authors attempted to induce recall of a fear memory using photo-activation. They used an AAV vector to transfect ChR2 under the control of a tetracycline response element promoter (tetO:ChR2) into mice carrying the tetracycline transactivator gene under the control of a c-Fos promoter (c-Fos:tTa). This arrangement allowed the researchers to externally suppress ChR2 expression, and then selectively allow expression during the presentation of a novel fear stimulus. The idea was that transcription of the immediate early gene, c-Fos would occur only in active cells of the hippocampus during presentation of the fear stimulus, which would then lead to transactivation of the ChR2 gene. Subsequently, the researchers showed that photo-activation of the ChR2-expressing population of cells resulted in freezing behavior of the mouse – indicative of fear memory recall. In addition, activation of ChR2 in cells active during presentation of non-fearful stimuli did not cause freezing behavior during photo-stimulation. This extremely novel result has important ramifications for the understanding of memory encoding, and raises ideas for many future studies on the role of such hippocampal “engrams” (Liu et al., 2012).

Studies from primate model systems are also invaluable for our understanding of human neurophysiology and for looking toward future clinical applications of optophysiology. Jazayeri et al. (2012) investigated vision and saccadic eye movements in rhesus monkeys, seeking to achieve optogenetic control of complicated primate behavior where past studies had encountered difficulties. Rhesus monkeys naturally orient their vision toward flashed stimuli. Thus, the researchers transfected areas of visual cortex (V1) with ChR2 using an AAV vector in an attempt to induce photo-controlled saccades. They found that photo-stimulation of the transfected region induced a large proportion of saccades in the direction corresponding to the receptive field of the region. They speculated that their success at inducing this visuomotor activity was due to the choice of V1 as the stimulation target (Jazayeri et al., 2012). While V1 circuitry may be primed to respond to unknown stimulation patterns, visuomotor areas may require specific patterns of input that studies using wide-field light activation are unable to deliver.

### NEURAL CIRCUITS AND NETWORKS

Here, we present several studies that highlight the potential of optophysiological studies on the function of neural networks and pathways. Sohal et al. (2009) developed the DIO strategy discussed above with an AAV vector to transfect various mouse cortical neuronal populations with ChR2 or eNpHR to study the role of parvalbumin (PV)-positive cortical interneurons in gamma-frequency oscillations of brain activity. They noted that past studies had suggested gamma-frequency activity may play a neuromodulatory role in information processing in the cortex. Interestingly, they found that inhibiting PV interneurons decreased, while stimulating cortical pyramidal neurons increased, the power of gamma-frequency oscillations *in vivo *(Sohal et al., 2009). The authors also showed that driving PV interneurons *in vitro* in brain slices increased the observed gamma-frequency oscillations in the preparation. Electroencephalography (EEG) studies have attempted to identify behaviors that are commonly associated with activity in specific frequency bands (Lutz et al., 2004). By drawing a link between a cell type and a previously extensively characterized oscillatory mode, the research helps to increase the relevance of EEG studies to behavior and disease.

Subsequent to this study, Piña-Crespo et al. (2012) produced embryonic stem-cell derived neurons, transfected them with ChR2 and NpHR, and transplanted them into cultured hippocampal slices. Using electrophysiology measurements with a multi-electrode array, they showed that light activation of the transplanted neurons could induce high-frequency oscillatory activity *in vivo* (Piña-Crespo et al., 2012). The researchers speculated on the therapeutic potential of such a transplant in an individual with disruptions in this oscillatory activity. Furthermore, this study demonstrates the effectiveness of combining electrophysiology with optophysiology.

## POTENTIAL CLINICAL APPLICATIONS

### MOVEMENT DISORDERS

Movement disorders of the basal ganglia pathway are numerous and complex, and none yet have a completely defined pathophysiology. The continuing trend of experiments seeking to explore this circuitry and its deficits during disease through targeted optophysiology manipulation may provide insight into both the neurotypic and pathologic brain. Indeed, several studies have already shown that optogenetic intervention can restore normal motor function in animal models of nigrostriatal degeneration (Gradinaru et al., 2009; Kravitz et al., 2010; Peppe et al., 2012).

One example comes from the research of Gradinaru et al. (2009), who sought to understand the reason that deep-brain stimulation (DBS) of the sub-thalamic nucleus (STN) can be beneficial for symptoms of Parkinson’s disease. The authors found that neither NpHR-mediated inhibition nor ChR2-mediated excitation of the STN produced an improvement of behavioral symptoms in rats with lesions to the nigrostriatal pathway. Interestingly, they did find that high-frequency ChR2-mediated stimulation of the afferent fibers to the STN produced a dramatic and reversible improvement of Parkinsonian symptoms, a result with clinical relevance that may guide the targeting of future DBS surgeries and other efforts at reducing symptoms.

Another study by Kravitz et al. (2010) also looked at basal ganglia pathways in Parkinson’s disease, but focused on different components of the pathway. They used an AAV vector to selectively transfect DIO ChR2 into mice expressing Cre recombinase in select populations of basal ganglia neurons. In this manner, they produced selective expression of the light-gated channel in only “direct” or “indirect” motor neuron pathways. They then showed that selective activation of indirect pathway neurons in the basal ganglia produced Parkinsonian-like motor behavior, while activation of direct pathway neurons produced the opposite effect. This result supported past theories about the roles of these two neuronal populations in motor control and Parkinson’s disease (Kravitz et al., 2010). Furthermore, they showed that selective transfection and activation of direct pathway neurons reversibly restored all motor deficits in a mouse model of Parkinson’s disease. Their results provide powerful support for the current model of Parkinson’s-induced basal ganglia degeneration and suggest a future of optogenetic DBS therapy with potentially better target specificity than current treatment efforts (Peppe et al., 2012).

Other optophysiological studies of movement disorders have focused on cell-replacement techniques. Tønnesen (2011) investigated the integration and subsequent functional state of stem cell-derived neural grafts with ChR2 and NpHR into striatal slices from a mouse model of Parkinson’s disease. The researchers used optical stimulation and inhibition of these transplanted neurons to show that the cells were capable of exhibiting normal synaptic activity. Their results support an exciting potential for application of optophysiology studies to uncover therapies for treatment of human Parkinson’s disease symptoms.

### SEIZURES AND EPILEPSY

Optically responsive protein tools may find important usage in the treatment of seizures and epilepsy. Current therapeutic approaches often rely on the use of broad-acting drugs or non-specific closed-loop electrical intervention, whereas an optophysiological treatment could have reduced peripheral side effects. A number of studies on varied animal models of epilepsy have already shown the potential of this research avenue (Bentley et al., 2013; Kokaia et al., 2013).

In their investigation of the cause of epilepsy subsequent to cortical trauma, Paz et al. (2013) found that inducing cortical stroke in rats leads to increased excitability of thalamocortical projections, as well as epileptiform activity in the thalamus. To probe the importance of these neurons in the observed epileptic phenotype, they stereotaxically injected an AAV vector containing eNpHR3.0 under the control of the CamK2a promoter, and found that inhibition of these thalamic neurons during a seizure can interrupt an ongoing seizure (Paz et al., 2013). Similarly, Sukhotinsky et al. (2013) studied the role of hippocampal pyramidal neurons in a rat lithium–pilocarpine model of epilepsy. Like Paz et al. (2013), they also used stereotaxic injection of an AAV vector containing eNpHR3.0 under the control of CamK2a promoter. They found that inhibition of the targeted hippocampal neurons caused a modest delay in the onset of status epilepticus. They noted that their single hemisphere experiment may have limited the effectiveness of their treatment, and that a dual hemisphere approach may have greater success (Sukhotinsky et al., 2013). These results support the exploration of regional inhibition in the prevention of epileptic seizures.

Only very recently have researchers explored the possibility of using optogenetic excitation of inhibitory interneurons as a method of blocking epileptiform activity. Krook-Magnuson et al. (2013) studied a mouse model of temporal lobe epilepsy (TLE) produced by unilateral dorsal hippocampal injections of kainate. They note that TLE provides an appealing target for therapeutic intervention because the onset of global epileptiform activity is generally preceded by a period of localized abnormal activity. In this way, there is an opportunity for the development of a closed-loop device – a device which detects and disrupts abnormal activity during the development of a seizure. The researchers used CamK2a:Cre to drive NpHR expression in excitatory neurons, or Parvalbumin:Cre to drive ChR2 expression in inhibitory neurons. They found that inhibition of *excitatory* neurons in the hemisphere ipsilateral to the kainate injection significantly interrupted seizures. Importantly, they found that excitation of *inhibitory* neurons using ChR2 on either the ipsilateral or the contralateral hemisphere also significantly interrupted seizures (Krook-Magnuson et al., 2013). Altogether, this important research provides strong support for the use of optogenetics in the treatment of seizures and epilepsy.

### VISION DISORDERS

Diseases of vision are also a potential area for clinical application of optophysiology. Indeed, this may be a natural application of optogenetic tools, as many of these proteins used originated in visual systems. Retinitis pigmentosa (RP) is a genetic disease that causes sequential dysfunction of rod and cone photoreceptor cells, leaving afflicted patients with intact but inactive cone cells. Busskamp et al. (2010) demonstrated the potential for functional recovery through optogenetic intervention by transfecting inactive cone cells with eNpHR3.0 using an AAV vector in a mouse model of RP. Since light normally hyperpolarizes photoreceptors in vertebrates, the authors could induce behavioral responses by triggering hyperpolarization of the transgenic cells, showing that some of the connectivity necessary for normal function remains after the cells become inactive. They also applied this technique to *ex vivo* human retinal tissue and successfully induced photocurrents. More recently, expression of ChR2 in mouse retinal ganglion cells combined with advanced retinal prosthetics has produced remarkable results on restoring vision in blind mice (Nirenberg and Pandarinath, 2012).

### HIGH-THROUGHPUT *EX VIVO* STUDIES

All-optical manipulation and measurement of cultured tissue may also hold promise as a high-throughput drug screening platform. One of the fastest growing areas of current clinical research is the creation of systems which may provide an informative model of neurodevelopmental disorders such as autism spectrum disorders (ASDs) or neurodegenerative disorders such as amyotrophic lateral sclerosis (ALS). The inaccessibility of nervous tissue from persons with neurogenetic disorders has been a long-standing obstacle for the study of these diseases. Recent advances in transcriptomic analysis suggest that expression data will be very valuable in characterizing disease states and evaluating potential therapeutics (Schmid et al., 2012). However, optophysiology can provide a direct, scalable cellular phenotype in addition to other readouts. The Cohen Lab at Harvard has demonstrated the capability to screen anti-arrhythmogenic drugs on induced pluripotent stem cell-derived cardiomyocytes (Adam Cohen, personal communication). They recently demonstrated the ability to discriminate drugs with pre-defined toxicity through optical measurements of drug-induced changes in conduction characteristics of cardiac action potentials. This model for an *ex vitro* drug-screening platform is appealing for its scalability and relative simplicity; much of the analysis can be automated by software, and the results are both reproducible and directly applicable to high-throughput drug evaluation.

## POTENTIAL TOOLS FOR THE OPTOPHYSIOLOGY TOOLBOX

While there are many optogenetic photoactuators and genetically encoded photosensors available, the search is still on for proteins that will allow greater temporal control and readout of a neuron’s activity. One exciting possibility for a fast voltage sensor could come from Tuberous electroreceptor cells. Weakly electric fish in the orders *Gymnotiformes* and *Mormyriformes* detect and encode changes in a projected electric field, including amplitude and frequency variation. These animals are sensitive to phase modulations of their own projected field as small as 100–300 ns, indicating an extremely high sensitivity in the frequency domain. If this sensitivity can be attributed to a sensory transduction protein, then there may be a possibility for coupling that protein’s voltage response to a fluorophore to produce a high frequency voltage probe (Kawasaki, 2009; Fortune and Chacron, 2011).

Another area for improvement results from the current spectral overlap of many of the current optophysiological tools. Many of the proteins engineered for photo sensing and actuation are based on the transduction mechanism of retinal pigments. This has been beneficial due to the well-characterized photocycle of opsins and the wide number of species from which they can be derived. However, another class of photosensitive proteins, called transient receptor potential (TRP) proteins has largely been unused in optophysiology. These TRP channels may have a unique and favorable set of properties as the basis for both sensors and actuators. Many subfamilies are sensitive to photons in infrared wavelengths, and function as the basis of thermoreceptive pit organs in reptiles, vision in some insects, and skin thermoreception in some mammals (Gracheva et al., 2010). Their distinctly shifted absorption spectra and different phototransduction mechanism may make them useful for increasing the variety of tools available for optophysiology.

## CONCLUSION

The cell- and tissue-specific expression of transgenic proteins and the tremendous library of past electrophysiology research decoding cellular events and activity has set the stage to address large, integrative questions on topics such as memory, emotion, attention and consciousness. Current optophysiological research continues to advance these questions and improve the technologies necessary to support the use of genetically encoded photosensors and optogenetic photoactuators, often concurrently (e.g., Akerboom et al., 2013). Innovations include the optics necessary for light-stimulation and recording, the software necessary for analysis of data produced, and the gene delivery methods necessary for spatiotemporal control of expression of transgenic proteins. Due to the rapid development of the field and the numerous opportunities for improvement of each component, optophysiology represents an exciting field of neuroscience with great potential for basic science and clinical research.

## Conflict of Interest Statement

The authors declare that the research was conducted in the absence of any commercial or financial relationships that could be construed as a potential conflict of interest.
